# The evolving landscape of genetic biomarkers for immunotherapy in primary and metastatic breast cancer

**DOI:** 10.3389/fonc.2025.1522262

**Published:** 2025-03-13

**Authors:** Liang Jin, Zijian Yang, Wei Tang, Pengli Yu, Rongrong Chen, Yan Xu, Jun Zhang

**Affiliations:** ^1^ Breast Tumor Center, Sun Yat-Sen Memorial Hospital, Sun Yat-Sen University, Guangzhou, China; ^2^ Department of Breast and Thyroid Surgery, Peking University Shenzhen Hospital, Shenzhen, China; ^3^ Department of Breast Surgery, The First Affiliated Hospital of Guangzhou Medical University, Guangzhou, China; ^4^ Medical Department, Geneplus-Beijing, Beijing, China; ^5^ Department of Breast and Thyroid Surgery, Daping Hospital, Army Military Medical University, Chongqing, China; ^6^ Department of Thyroid and Breast Surgery, Shenzhen Qianhai Shekou Free Trade Zone Hospital, Shenzhen, China

**Keywords:** breast cancer, genomic profile, primary tumor, immunotherapy, PD-L1 amplification, TNBC

## Abstract

**Background:**

Major advances have been achieved in the characterization of primary breast cancer genomic profiles. Limited information is available on the genomic profile of tumors originating from different metastatic locations in recurrent/metastatic (R/M) breast cancer, especially in Asian patients. This study aims to decipher the mutational profiles of primary and R/M breast cancer in Chinese patients using next-generation sequencing.

**Methods:**

A total of 563 breast cancer patients were enrolled, and 590 tumor tissues and matched peripheral blood samples were collected and subjected to targeted sequencing with a panel of 1,021 cancer-related genes. The mutation spectrum, DNA damage response (DDR) genes, commonly altered signal pathways, and immunotherapy-related markers were compared between primary and R/M breast cancer. The molecular differences between our cohort and the Memorial Sloan Kettering Cancer Center (MSKCC) dataset were also explored.

**Results:**

A total of 361 samples from primary and 229 samples from R/M breast cancer were analyzed. *BRCA2, ATRX*, and *ATM* were more frequently observed in R/M lesions among the 36 DDR genes. An *ESR1* mutation and *PD-L1* and *PD-L2* amplification were enriched in R/M breast cancer (all *p*<0.05). Compared with the MSKCC dataset, we recruited more patients diagnosed at age 50 or younger and more patients with triple-negative breast cancer (TNBC) subtypes. The TNBC patients in our dataset had a higher percentage of *PD-L1* amplification in metastasis tumors (*p*<0.05).

**Conclusions:**

This study revealed the distinctive mutational features of primary and R/M tumors in Chinese breast cancer patients, which are different from those from Western countries. The enrichment of *PD-L1* amplification in metastatic TNBC indicates the necessity to re-biopsy metastatic tumors for immunotherapy.

## Introduction

Breast cancer (BC) is the most common cancer and the fifth leading cause of cancer deaths in women in China (1). Breast cancer is divided into molecularly distinct subtypes based on hormone receptor (HR) and human epidermal growth factor-positive (HER2) status and these lead to different clinical outcomes and choice of therapies (2). Large-scale genomic characterization of breast cancer has established the genomic landscape and has contributed to the current understanding of complex mutational features among different breast cancer molecular subtypes (3–6). The genomic information of primary breast cancer and that of recurrent/metastatic (R/M) tumors have also been reported in several studies using patients from Western countries and provide pivotal insights in identifying genomic drivers associated with metastasis and targetable mutations in metastatic tumors. However, breast cancer patients in China have a much younger age at onset of breast cancer and a distinct molecular subtype distribution compared with patients in Western countries (7). It remains unclear whether the genetic changes in metastatic breast cancer in China are different from those in Western countries. However, the genetic landscape between primary and R/M tumors in Chinese breast cancer patients has not yet been characterized.

Currently, immune checkpoint inhibitor (ICI)-based therapies have shown remarkable promise for early-stage or advanced triple-negative breast cancer (TNBC). Compared with hormone receptor-positive (HR+) breast tumors, both TNBC and HER2+ tumors have a higher degree of stromal and intratumoral tumor-infiltrating lymphocytes (TILs) (20% and 16% of cases, respectively) (8, 9) and high immune-related gene expression (10). Moreover, TNBC has a relatively higher tumor mutation burden (TMB) than other breast cancer subtypes and higher rates of cell surface PD-L1 expression. Thus, currently, PD-1/PD-L1 targeted immunotherapy is mostly used in this subset of patients (11–13).

Indeed, checkpoint inhibition with the anti-PD1 antibody pembrolizumab has been approved for advanced-stage PD-L1-positive TNBC due to improved outcomes when combined with frontline chemotherapy (14). However, the IMpassion131 study, with a combination of paclitaxel and the PD-L1 inhibitor atezolizumab, failed to improve progression-free survival (PFS) or overall survival (OS) in advanced TNBC patients (15). Landmark phase III trials tested immunotherapy in the early-stage neoadjuvant setting KEYNOTE-522 trial indicated that addition of pembrolizumab to chemotherapy improved the pathological complete response (pCR) rate (16). Similarly, IMpassion031, in which atezolizumab was combined with chemotherapy, resulted in improved pCR rates compared to chemotherapy alone, regardless of PDL1 status (17). In spite of the great progress of immune checkpoint inhibitors in TNBC, not all patients respond to immunotherapy and there is a strong need to identify prognostic and predictive biomarkers.

Several studies have demonstrated that the PD-L1 status is insufficient for identifying responder patients (18). In the neoadjuvant setting of TNBC, immunotherapy seems to provide benefits regardless of the PD-L1 status (19). Moreover, a recent analysis of the Tumor Cancer Genome Atlas (TCGA) revealed that PD-L1 positivity is only weakly associated with immunotherapy efficacy (20). In breast cancer, especially TNBC, there were discrepancies in its expression between primary tumors and metastatic sites. Primary tumors tend to have higher rates of PD-L1 expression compared to metastatic disease, especially in the liver, skin, and bones whilst PD-L1 expression in lung and lymph node metastases was comparable to that of the primary site (21) (22).

The KEYNOTE-158 trial showed that patients with previously treated advanced-stage solid tumors responded better to pembrolizumab monotherapy if their tumors had a high TMB in a myriad of primary tumor types including thyroid, anal, cervical, biliary, and endometrial (23). Data from GeparNuevo, a phase II neoadjuvant trial (24), and the TAPUR study (25) showed that TMB and immune cell infiltration could serve as independent predictors of response to immune checkpoint inhibition in early or metastatic TNBC. Indeed, a high TMB is more likely to be associated with a deficiency of the MMR pathway or homologous recombination repair system, genetic alterations in DNA polymerase genes (POLE/POLD1), and the APOBEC mutation signature (26). Of note, compared to early BC, more advanced tumors generally display a higher TMB and less abundant TIL levels (27, 28). However, ICIs are more effective in treating TNBC when given early in the course of the disease, which was generally attributed to immune escape mechanisms emerging during the progression of the disease (29), and less was known about the contribution of the evolving mutation profile.

TNBC often involves high lymphocyte infiltration and the measurement of tumor infiltrated lymphocytes (TILs) has been proposed as a surrogate marker of the adaptive immune response against neoplastic cells (30) (31). Data from KEYNOTE-086, a phase II study, revealed that TIL levels can predict response to immunotherapy, particularly in the first-line setting (32, 33). In the metastatic setting, a higher density of CD8+ TILs was associated with increased PFS and OS in the IMpassion130 trial (34). Interestingly, the phase II FUTURE-C-Plus trial showed that patients with CD8- and/or PD-L1- positive tumors benefited more from immunotherapy while a *PKD1* somatic mutation indicated worse progression-free and overall survival (35). This indicates that mutation profiles may also contribute to the diverse response to immunotherapies.

Several studies have also demonstrated that BRCA1/2 mutation was associated with an immune activation signature and might predict immunogenicity in BRCA1/2-deficient BC, including TNBC (36, 37). Recently, the phase II I-SPY2 trial showed that the combination of PD-L1 inhibitor, PARP inhibitor, and standard paclitaxel neoadjuvant chemotherapy has superior efficacy over standard neoadjuvant chemotherapy in HER2-negative breast cancer and in a subset of high-risk HR-positive/HER2-negative patients (38). Besides *BRCA1/2*, approximately 20% of TNBC have a loss of *PTEN*, leading to a more immunogenic drive, however, its role in immunotherapy is still controversial (11, 39).

To better understand the mutation spectrum between primary and R/M tumors in Chinese breast cancers, we retrospectively analyzed the genomic data of 600 samples from 570 Chinese patients with various molecular and histological subtypes of early-stage and R/M breast cancer to elucidate their mutational landscape. Furthermore, we also performed a subgroup analysis by metastatic lesion to discover significantly altered genes and compared the genomic data of samples originating from the same metastasis lesions between our cohort and the Memorial Sloan Kettering Cancer Center (MSKCC) breast cancer dataset.

## Materials and methods

### Patients

Between May 2017 and December 2021, a total of 563 patients diagnosed with malignant breast cancer were retrospectively enrolled in this study. All patients provided written consent for genetic analysis and the protocol was approved by the Ethics Committee of Sun Yat-sen Memorial Hospital (2019-KY-073). ER/PR positivity in the immunohistochemistry testing was indicated with a cut-off of equal or higher than 1% positively stained cells (40). HER2 status was defined by an immunohistochemistry score of 0 or 1 or a lack of *HER2* amplification (ratio <2.2) demonstrated by FISH analysis.

### Sample processing and DNA extraction

Tumor DNA was extracted from formalin-fixed, paraffin-embedded (FFPE) tumor tissue specimens using the ReliaPrep FFPE gDNA Miniprep System (Promega). Matched germline genomic DNA was isolated from peripheral blood lymphocytes (PBL) using the QIAamp DNA Blood Mini Kit (Qiagen). The concentration and fragment length of DNA were determined using an Agilent 2100 Bioanalyzer (Agilent Technologies, Inc.).

### Library construction and next-generation sequencing

Germline genomic DNA and tumor DNA were sheared into fragments at a 200 to 250 bp peak with a Covaris S2 ultrasonicator (Covaris, Inc), and indexed next-generation sequence (NGS) libraries were prepared using an NEBNext Ultra DNA Library Prep Kit for Illumina (NEB). For tumor genomic and matched germline DNA libraries, a previously reported custom-designed panel (Integrated DNA Technologies, Inc.) covering ∼1.5 Mbp of the genome and 1,021 cancer-related genes was used for hybridization enrichment (41). The indexed libraries were sequenced on a Gene+Seq-2000 sequencing system (GenePlus-Suzhou).

### Raw data processing tumor somatic variant calling

The sequenced reads were mapped to the reference human genome (GRCh37) using the default parameters in BWA version 0.7.12 after removing adaptor and low-quality reads. Local realignment around single nucleotide variants (SNVs) and small insertions and deletions (Indels), and quality control assessment, were performed using GATK (version 3.4.46; Broad Institute). Genomic alterations, including SNVs, Indels, copy number alterations (CNA), and gene fusions/rearrangements, were detected with GATK, MuTect (version 1.1.4) and BreakDancer, respectively. PBL DNA was used as a control to identify somatic variations from germline variations.

### Statistical analysis

An MSK dataset (MSKCC, Cancer Cell 2018) was downloaded from the cBioPortal website (https://www.cbioportal.org/study/summary?id=breast_msk_2018, accessed on 11 May 2022). The chi-square test or Fisher’s exact test was used to analyze frequencies of genetic alterations in different groups. As for gene mutation rates among the three subgroups, a chi-square test was performed at first. Then, if *p*<0.05, Fisher’s exact test and the Bonferroni correction were conducted to analyze the difference between two subtypes. Statistical processing was performed with SPSS version 23 (SPSS Inc., Chicago, IL, USA), and *p* < 0.05 (two-sided) was considered significant.

## Results

### Characteristics of the study population

In this study, 590 tumor tissues and matched peripheral blood samples were collected from 563 breast cancer patients (**Table 1**), with 25 patients having two or three tumor samples. Briefly, all the patients were female, and 68.6% were diagnosed at age 50 or younger. Among these 563 patients, 7 patients (1.2%) were diagnosed at stage 0, 84 patients (14.9%) at stage I, 234 patients (41.6%) at stage II, 176 patients (31.3%) at stage III, and 56 patients (9.9%) at stage IV. A total of 361 (61.2%) samples originated from primary breast cancer and 69 (11.7%) samples originated from recurrent breast cancer. The remaining 160 (27.1%) samples represented metastases obtained from lymph nodes (n = 51), liver (n = 38), chest (n = 21), lung (n = 20), brain (n = 12), and distant metastases from other sites (n = 18). The molecular subtypes of tumors include HR+/HER2- (36.8%), HR+/HER2+ (7.1%), HR-/HER2+ (14%), and TNBC (42.1%), based on pathological immunohistochemical staining of sequenced samples.

**Table 1:** Clinicopathological characteristics in breast cancers.

Variable	No.	(%)
Age		
≤50 years	386	68.6%
>50 years	177	31.4%
TNM stage		
0	7	1.2%
I	84	14.9%
II	234	41.6%
III	176	31.3%
IV	56	9.9%
Unknown	6	1.1%
Molecular subtype		
HR+/HER2-	207	36.8%
HR+/HER2+	40	7.1%
HR-/HER2+	79	14.0%
TNBC	237	42.1%
Sample location		
Primary breast	361	61.2%
Recurrent breast	69	11.7%
Lymph node	51	8.6%
Liver	38	6.4%
Lung	21	3.6%
Chest	20	3.4%
Brain	12	2.0%
Other distant metastases	18	3.1%

HR, hormone receptor; HER2, human epidermal growth factor; TNBC, triple-negative breast cancer.

### Molecular landscape of somatic genomic alterations in breast cancer

**Figure 1:**
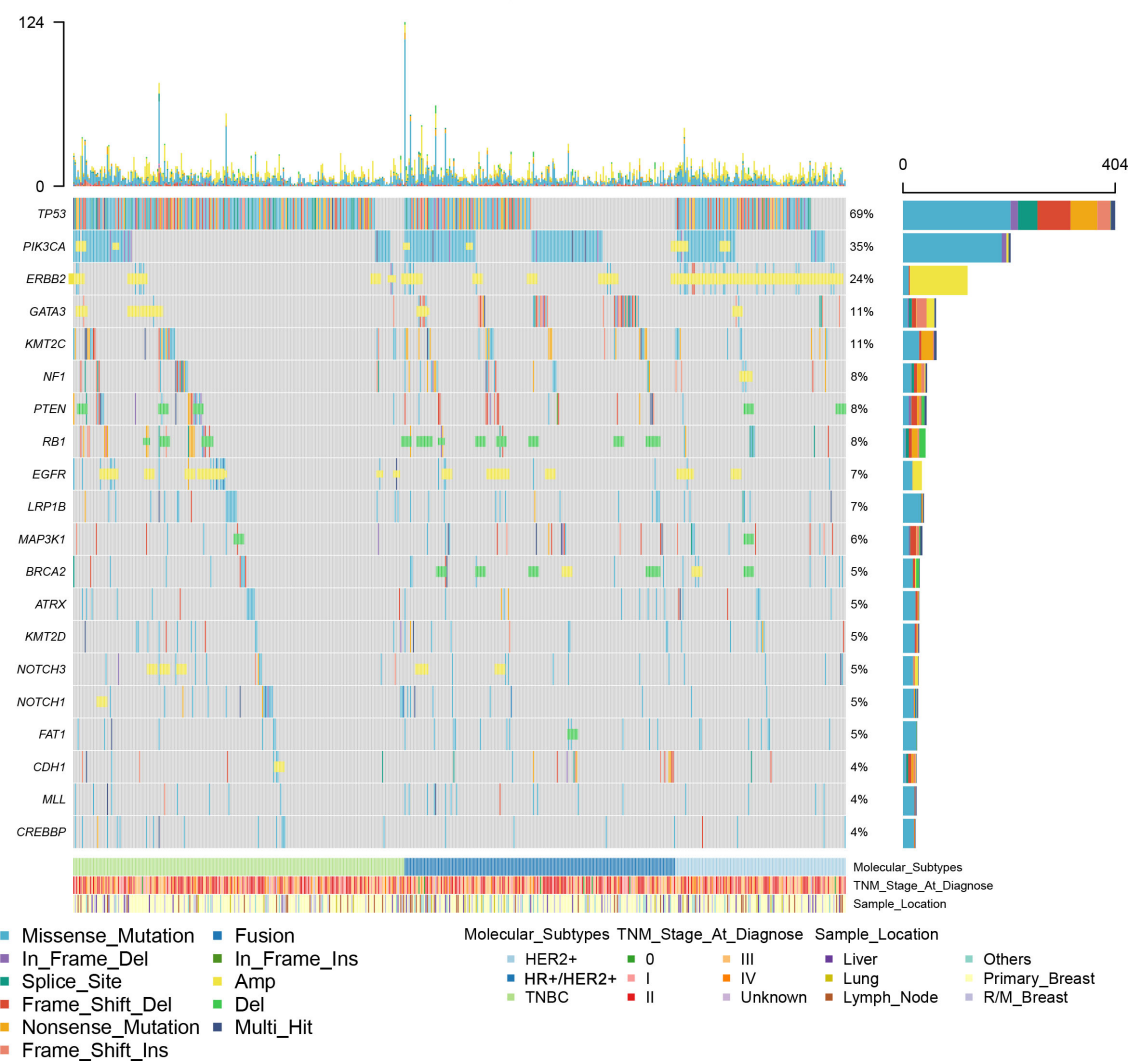
A summary of the genomic characteristics of 590 samples from Chinese patients with breast cancer. Oncoprint showed genetic changes with an incidence of more than 4%. According to HR and HER2 status, the tumor samples were categorized into HR+/HER2- (n = 215), HER2+ (n = 128), or triple-negative (n = 247). Clinicopathological features are annotated at the bottom. Amp, copy number amplification; Del, copy number deletion; HR, hormone receptor; HER2, human epidermal growth factor.

All 590 breast cancer samples from 563 patients were tested using the 1,021-gene NGS panel. A total of 5,883 somatic alterations were detected in the tumor samples of these patients, including 4,020 SNVs/Indels, 1,814 CNVs, and 49 SVs. The number of alterations detected in each sample ranged from 0 to 124 with a median of 8. No somatic alteration was observed in four tumor samples (4/590, 0.7%). The most common mutational type was a missense mutation (51.1%), followed by CN gain (29.4%), frameshift mutation (6.7%), and nonsense mutation (5.4%). *TP53* was the most frequent somatic alteration observed in breast cancer patients and the prevalence of this mutation was 69% (**Figure 1**). Other genomic variations composed of SNVs/Indels and CNVs with an incidence higher than 10% in all the samples include *PIK3CA* (35.4%), *MYC* (26.1%), *ERBB2* (24.6%), *CDK12* (13.5%), *CCND1* (11.6%), *KMT2C* (11.0%), *MCL1*(10.6%), and *GATA3*(10.5%).

### Comparison between primary and relapse/metastasis breast samples

**Figure 2:**
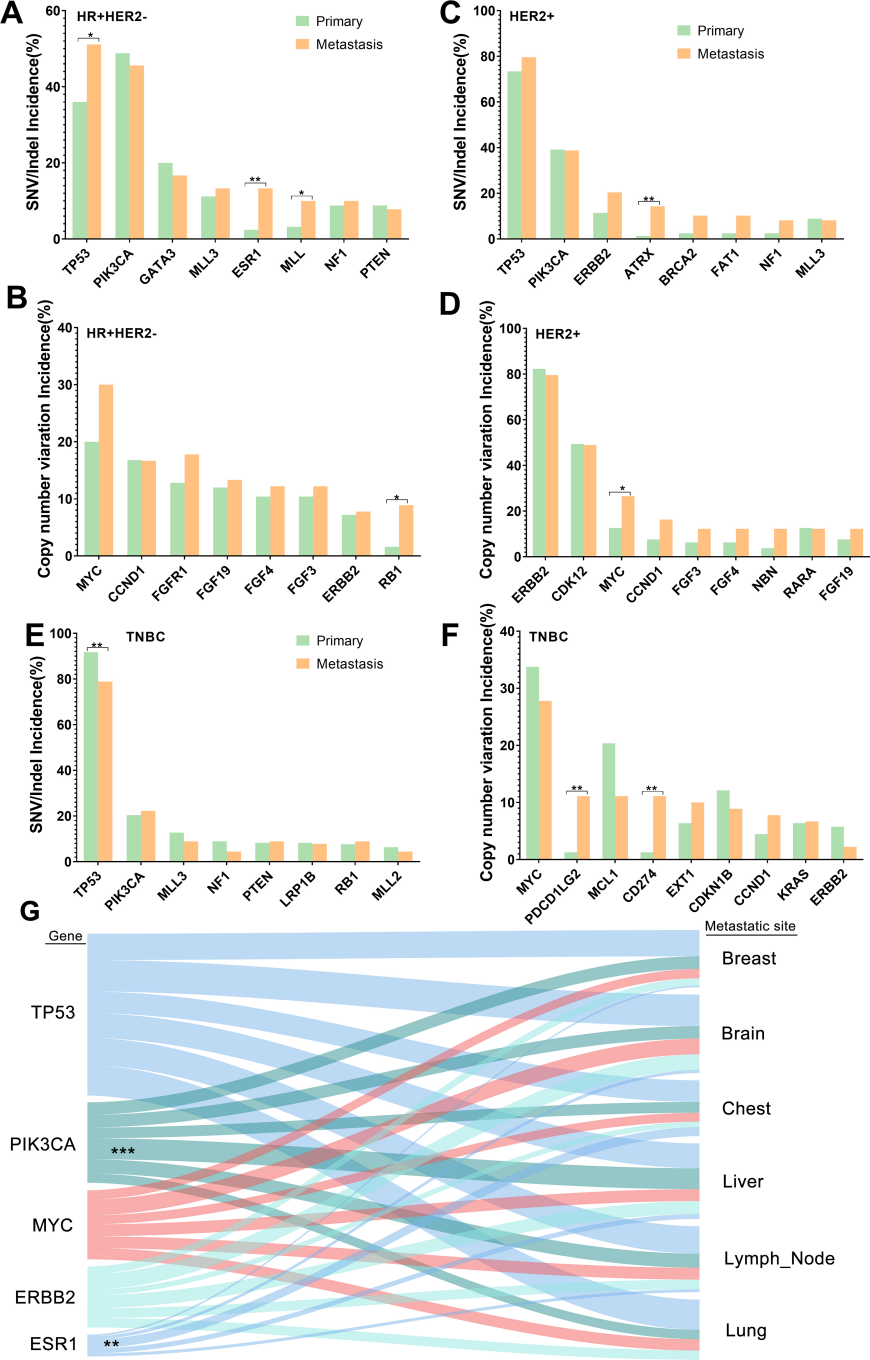
Commonly mutated genes in primary and R/M breast samples. **(A, B)** Top altered SNVs/Indels and CNVs in HR+/HER2- primary and R/M breast samples. **(C, D)** Top altered SNVs/Indels and CNVs in HER2+ primary and R/M breast samples. **(E, F)** Top altered SNVs/Indels and CNVs in TNBC primary and R/M breast samples. **(G)** Genomic alterations (left) and their association with different organ sites of metastases (right). Line thickness corresponds to the frequency of mutations arising in the indicated metastatic site. Shading identifies the relationships between genes and metastatic sites. Statistically significant associations are shown as asterisks. R/M, recurrent/metastasis; SNV, single-nucleotide variants; Indel, insertion and deletion, CNV, copy number variation; HR, hormone receptor; HER2, human epidermal growth factor; TNBC, triple-negative breast cancer. * denotes *p*<0.05, ** denotes *p*<0.01, *** means p<0.001..

We further assessed the distribution of mutational signatures between primary breast cancer and R/M breast cancer in order to better understand which mutational processes drive tumor recurrence/metastasis. The observed common SNVs between primary and R/M breast cancer are shown in **Figure 2A**. The top three most commonly mutated genes were *TP53*, *PIK3CA*, and *KMT2D* in both groups. *ESR1* was the only gene among the top 14 genes that had different mutation rates between primary (0.8%) and R/M tumors (7.4%) (*p*<0.05). In primary breast cancer, an analysis of molecular subtypes demonstrated that *TP53* mutations were more frequent in HER2+ and TNBC breast cancer than in other subtypes (*p*<0.05, **Figure 2B**). Moreover, *PIK3CA* mutations were enriched in HR+ patients, *GATA3* mutations were more common in HR+/HER2- patients, and *ERBB2* was frequently mutated in HER2+ patients (all *p*<0.05). *PTEN* was exclusively mutated in HR+/HER2- and TNBC tumors. In R/M breast cancer, the distribution of mutations in *TP53*, *PIK3CA*, *GATA3*, and *PTEN* were similar to those in primary breast cancer (**Figure 2C**), indicating these gene mutations have similar roles in distinctive molecular subtypes of both primary and R/M tumors.

The distribution of common CNVs between primary breast cancer and R/M breast cancer is shown in **Figure 2D**. The top three CNVs observed were *MYC*, *ERBB2*, and *CDK12* in both groups. The distribution of the top 14 CNVs was similar between the two groups. In primary breast cancer, an analysis of molecular subtypes demonstrated that *MYC* was more enriched in TNBC than HER2+ (33.7% vs 12.7%, *p*<0.01) (**Figure 2E**). Furthermore, *ERBB2* and *CDK12* amplifications were more likely to be observed in HER2+ patients. Furthermore, *CCND1* and *FGF3/4/19* were more frequently altered in HR+/HER2- tumors and *MCL1* and *CDKN1B* were more enriched in TNBC (all *p*<0.05). In the R/M breast cancer cohort, the distribution of mutations in *ERBB2*, *CDK12*, and *FGFR1* were similar to those in primary breast cancer. However, compared to primary tumors, the frequency of *FGF3/4/19* variations in R/M breast cancer was elevated in TNBC and HER2+ (**Figure 2F**).

Furthermore, we decided to identify mutations that reflected organotropism in their pattern of metastasis (**Figure 2G**). For example, *ESR1* mutations were associated with metastasis to the chest, while *PIK3CA* mutations were associated with liver metastasis.

### Commonly altered pathways between primary and R/M breast cancers

**Figure 3:**
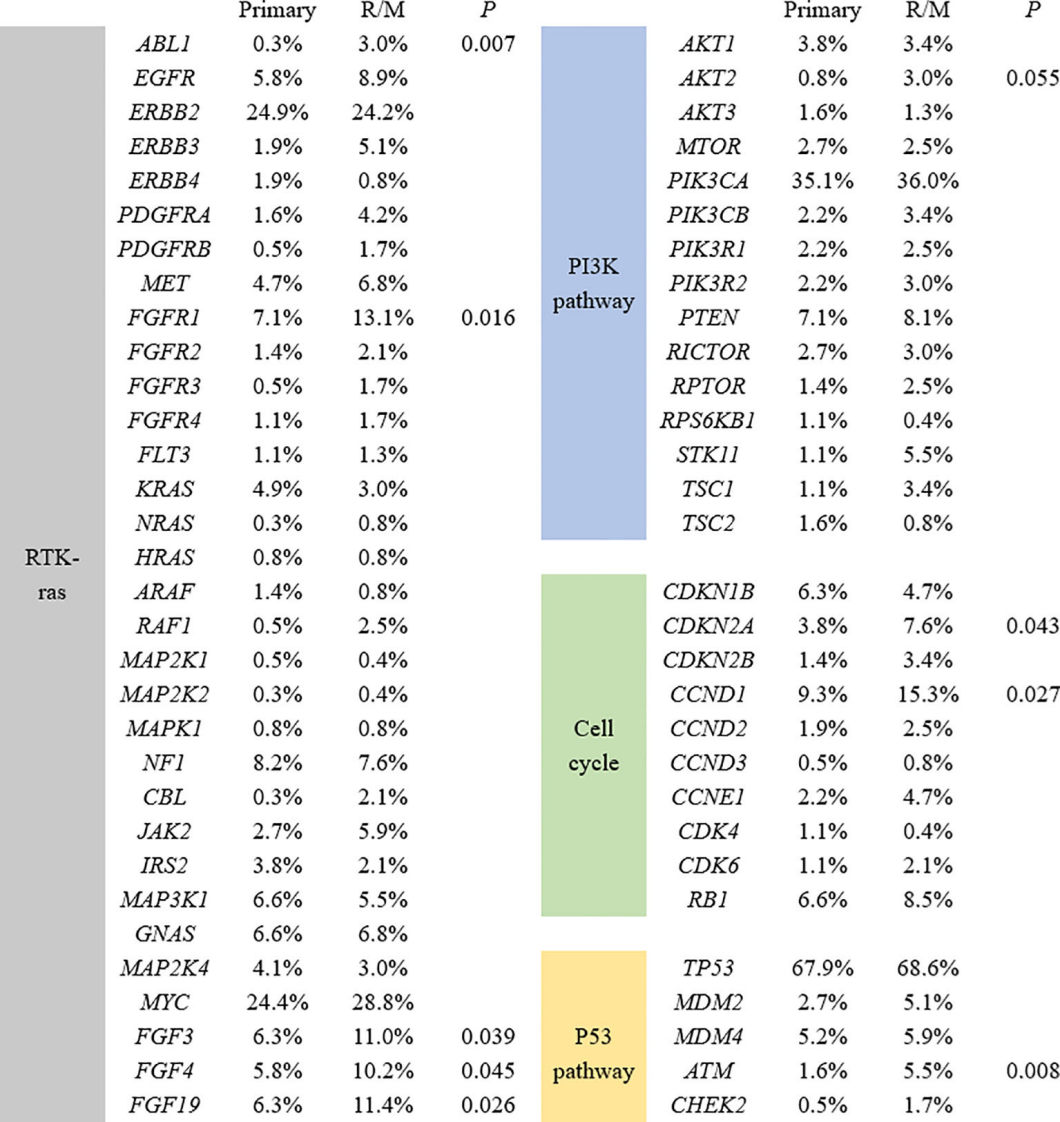
Significant differences in mutant genes between primary and R/M breast samples in four signaling pathways. R/M, recurrent/metastasis samples.

We further analyzed the key signaling pathways commonly mutated in all samples. The most common genomic alteration was the P53 pathway (72%), followed by the RTK/Ras/MAPK signaling pathway (66.4%), the PI3K-AKT pathway (54.4%), cell cycle control (28.8%), and the FGF superfamily (9.3%) (**Figure 3**). Among the genes belonging to the P53 pathway, ATM was the only gene that exhibited statistically different mutation frequencies between primary and R/M breast cancer. In the RTK/Ras/MAPK signaling pathway, the most commonly mutated genes were *ERBB2* (24.6%), *FGFR1* (9.5%), and *NF1* (8.0%). Compared to primary cancer, *ABL1*(0.3% vs 3.0%, *p*=0.007) and *FGFR1* (7.1% vs 13.1%, *p*=0.016) were more likely to be observed in R/M sites. The most frequently altered genes in the PI3K-AKT pathway were *PIK3CA* (35.4%), *PTEN* (7.5%), and *AKT1* (3.7%). No significant differences in the genes of the PI3K-AKT pathway were observed between primary and R/M breast cancer. Among the genes in the cell cycle pathway, *CCND1* (11.6%), *RB1* (7.3%), and *CDKN1B* (5.7%) were the top 3 most mutated genes. *CCND1* (15.3% vs 9.3%, *p*=0.027) and *CDKN2A* (7.6% vs 3.8%, *p*=0.043) exhibited higher mutation rates in R/M tumors than in primary tumors. Among the genes in the FGF superfamily, *FGF19*, *FGF3*, and *FGF4* were the most frequently mutated with an incidence of 8.3%, 8.2%, and 7.5%, respectively. R/M tumors more frequently harbored alterations in *FGF19* (11.4% vs 6.3%, *p*=0.026), *FGF3* (11.0% vs 6.3%, *p*=0.039), and *FGF4* (10.2% vs 5.8%, *p*=0.045) than primary tumors.

### Characteristics of somatic DNA damage response gene mutations between primary and R/M breast cancers

**Figure 4:**
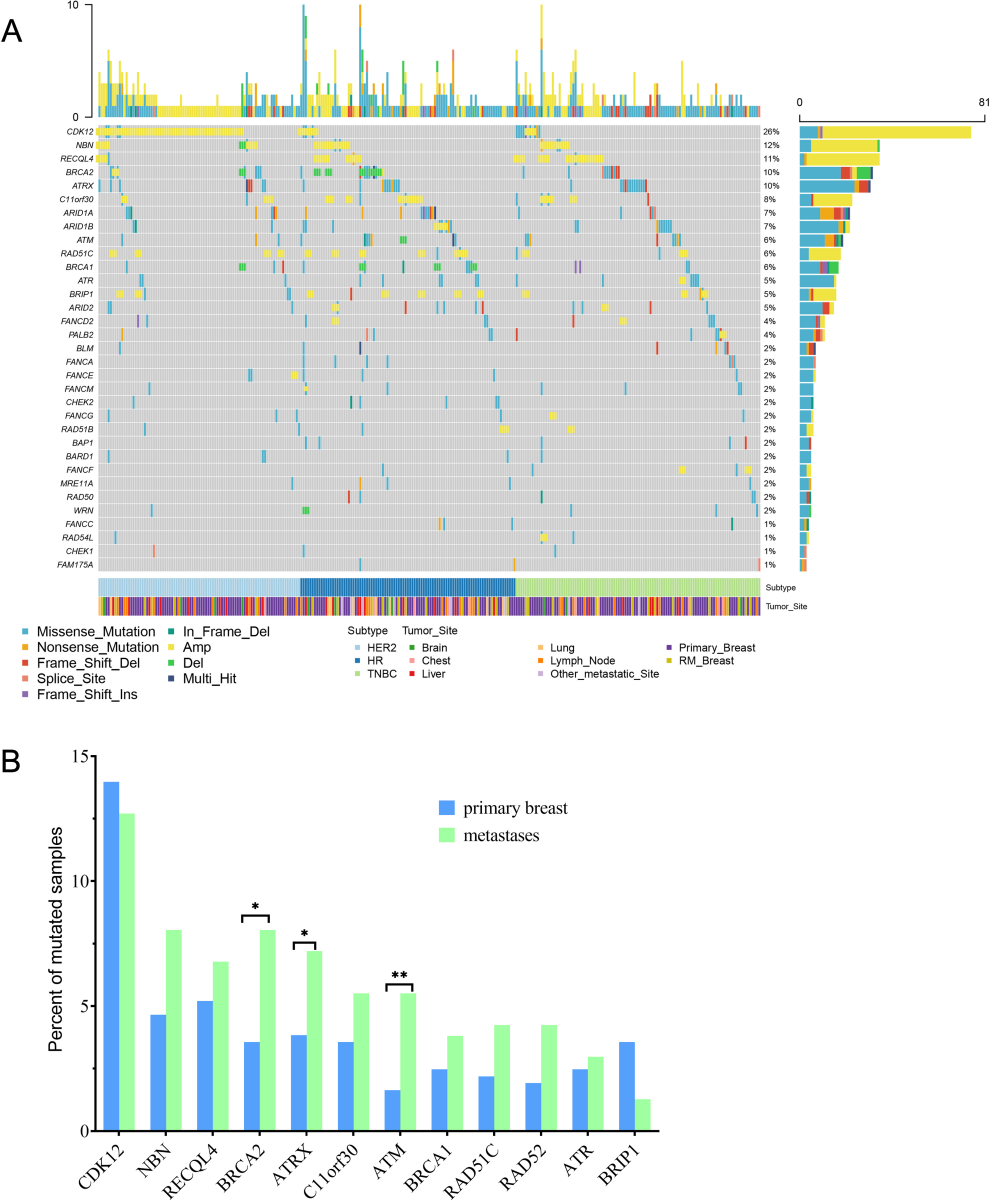
Comparison of 36 DDR genes between primary and R/M breast cancer. **(A)** Oncoprint showed genetic changes with an incidence of more than 1%. **(B)** Distribution of the top 12 mutated DDR genes between primary and R/M breast cancer. DDR, DNA damage repair; R/M, recurrent/metastasis; * denotes *p*<0.05, ** denotes *p*<0.01.

We next evaluated somatic alterations in 36 DDR genes covered by our NGS panels, including *ATM, ATR, ATRX BAP1, BLM, BARD1, BRCA1/2, BRIP1, CHEK1/2, CDK12, EMSY, ERCC1, FAM175A FANCA/C/D2/E/F/G/L/M, MRE11A, NBN, RAD50/51/52, RAD51B/C/D, RAD54L, PALB2, RECQL, RECQL4*, and *WRN*. We found 286 (47.6%) breast cancer patients had DDR gene mutations. There were 12 genes detected with more than 2.5% incidence (SNVs and CNVs) across all samples. (**Figure 4A**). The most frequently altered DDR gene in patients was *CDK12* (n =81, 13.5%). The mutation frequencies of *CDK12* in primary and R/M breast cancer were 14.0% and 12.7%. R/M breast cancer more frequently harbored alterations in *BRCA2*, *ATRX*, and *ATM* (*p*<0.05, **Figure 4B**). There were no significant differences in other DDR genes between primary and metastatic tumors.

### Immunotherapy-related markers between primary and R/M breast cancers

**Figure 5:**
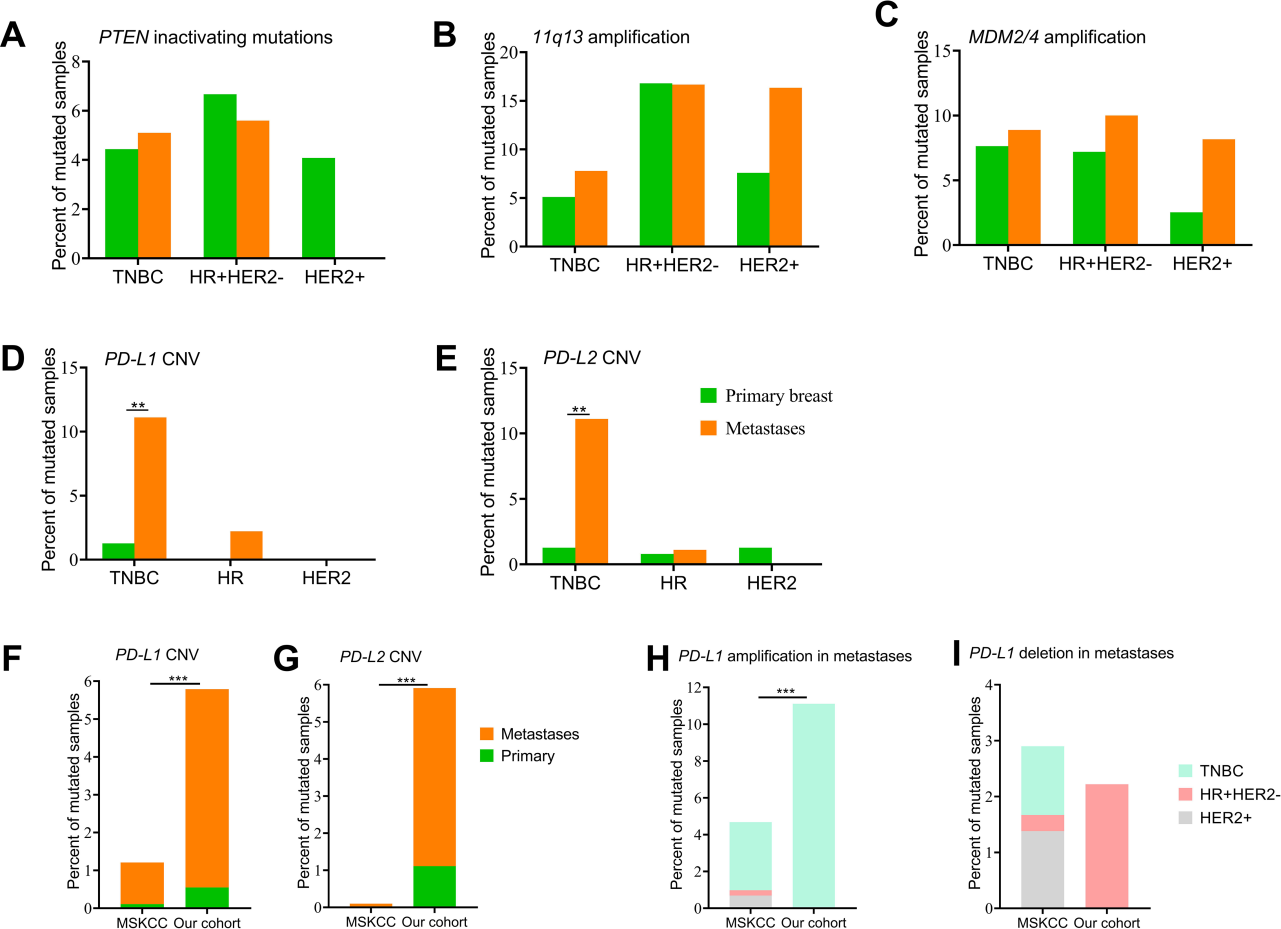
Genomic alterations of immunotherapy biomarkers. **(A)** *PTEN*-inactivating mutations between primary breast cancers and metastases. **(B-E)** Copy number variations of genes in the 11q13 region, *MDM2/4*, *CD274/PD-L1*, and *PDCD1LG2/PD-L2* between primary breast cancers and metastases. **(F, G)** Distribution of *CD274/PD-L1* and *PDCD1LG2/PD-L2* copy number variations between our cohort and the MSKCC dataset. **(H, I)** Number of *PD-L1* amplifications and deletions among the three subgroups between our cohort and the MSKCC dataset. CNV, copy number variation; HR, hormone receptor; HER2, human epidermal growth factor; TNBC, triple-negative breast cancer Del, deletion; Amp, amplification. ** denotes *p*<0.01, *** means p<0.001.

It is known that primary and R/M breast cancer have distinct immune microenvironments that impact their response to therapy. Thus, we investigated gene variations that are associated with the immunotherapy response. *PTEN*-inactivating mutations, amplification of *MDM2/4*, and amplification of the 11q13 regions (including *CCND1*, *FGF3/4/19*) were reported to be negative or hyperprogressive biomarkers of immunotherapy. The distribution of *PTEN*-inactivating mutations and *MDM2/4* amplification were similar between primary and R/M breast cancer among three subtypes (**Figures 5A, C**). Amplification of the 11q13 regions was frequently observed in patients with HR+HER2- and HER2+ breast cancer (**Figure 5B**). In addition, compared to samples with HR+HER2- and HER2+, *PD-L1* amplification was only observed in TNBC (**Figures 5D, H**). CN gain in *PD-L1* was detected in 1.3% (2/157) of primary TNBC and 11.1% (10/90) of metastasis TNBC samples. Similar to *PD-L1*, *PD-L2* CNVs were more often observed in metastasis TNBC (**Figure 5E**).

To better understand molecular differences among primary tumors and metastases between patients from China and Western countries, we obtained the clinical information and results of NGS survival in 1,918 samples from 1,756 breast cancer cases (MSKCC, Cancer Cell 2018), which was available on the cBioPortal website. Compared with the MSKCC dataset, we recruited more breast cancer patients diagnosed aged 50 years or younger and more patients with the TNBC subtype (**Supplementary Table 1**). The MSKCC dataset included 905 samples of primary breast cancer and 1,000 samples of metastasis disease (**Supplementary Table 2**). A total of seven *CD274/PD-L1* CN gains (0.4%, 7/1918) and five *PD-L1* CN deletions (0.3%, 5/1918) were identified, and only one sample originated from primary breast cancer with the HER2 subtype and the other samples were from metastatic sites. Among the 11 metastatic samples, four samples were of the TNBC subtype, four samples were the HR+/HER2- subtype, and three samples were the HER2+ subtype. Only one *PDCD1LG2/PD-L2* CNV was identified in a sample of the HR+/HER2- subtype (**Figures 5F, G**). Higher percentages of *PD-L1* and *PD-L2* CNVs were identified in metastatic samples in our cohort. We then explored *PD-L1* amplification and deletion among the three subtypes between the MSKCC dataset and our cohort and no significant difference was observed (**Figures 5H, I**).

## Discussion

In the present study, we explored the genomic characteristics of primary and R/M breast cancer in a large cohort of Chinese patients. Both tumor samples and matched blood specimens from each clinical participant were collected and tested for NGS profiles containing 1,021 cancer-related genes. Our study included patients with TNBC and HR+ disease. In our cohort, 99.3% (597/601) of the samples exhibited at least one mutation. *TP53* (68.2%), *PIK3CA* (35.4%), *MYC* (26.1%), and *ERBB2* (24.6%) were mutated in more than 20% of the cohort. In this subgroup alone, three genes were more commonly mutated in R/M tumors versus primary tumors. We compared the mutational spectrum, DDR genes, common signal pathways, and immunotherapy-related markers between primary and R/M tumors. R/M breast cancer more frequently harbored alterations in *BRCA2* and *ATM*. *PD-L1* and *PD-L2* amplifications were more likely to be observed in R/M sites in TNBC. Among the multiple genes involved in common altered signal pathways, *ABL1*, *FGFR1*, *CCND1*, *CDKN2A*, *ATM*, and *FGF3/4/19* were detected more frequently in R/M sites than in primary tumors. Subgroup analysis by metastatic location demonstrated chest wall metastases more frequently harbored alterations in *FGFR1*, while liver metastases more frequently had mutations in *CCND1* and *FGF3/4/19*.

In the present study, *TP53* was the most frequently occurring somatic mutation in all samples and the prevalence of this mutation was 68.2%. The mutation rate of *TP53* (53%, n=1314) was significantly lower in a previous study launched by the Fudan University Shanghai Cancer Center (FUSCC) (4), which also included Chinese patients with both primary and metastasis cancer. Compared with FUSCC (4), the MSKCC (42) and TCGA (43) datasets, which enrolled a larger number of HR+ patients, HR+ and TNBC patients were found in similar proportions (43.1% and 41.6%, respectively) to our cohort. The mutation rate of *TP53* was nearly 90% in TNBC in our cohort, which may have increased the overall *TP53* mutation frequency. In addition, the NGS profiles of 1,021 genes encompassed all introns and exons of *TP53* (44), while the FUSCC-BC panel may have omitted the introns of *TP53* (4). The *PIK3CA* frequency was approximately 48% in HR+ tumors, 31.9% in HR-/HER2+ tumors, and 20.4% in TNBC tumors, which were consistent with the mutation frequencies reported in a previous study (6).

We further systematically explored the differences between primary and R/M breast cancer through multiple aspects, including mutation enrichment, DDR genes, oncogenic pathway alterations, and immunotherapy-related markers. *ATM* was among the DDR genes and involved in the p53 pathway. A higher *ATM* mutation rate was observed in R/M tumors. This result was consistent with a previous study that showed that patients with *ATM* mutations develop intermediate- or high-grade disease and have a higher rate of lymph node metastasis (45). ATM has been widely identified as a promising drug target. Up to now, several ATM inhibitors have been developed by different companies and entered clinical trials (46). Due to the development of synthetic lethal targets, higher rates of DDR defects in R/M tumors may expand the subset of patients that derive benefit from PARP inhibitors and other DDR-targeting drugs in the clinic (46). The mutation rate of *TP53* in primary and R/M tumors in HR+/HER2- patients was 58.5% and 35.5%, respectively. *ESR1* was also enriched in HR+ tumors from metastasis sites. Two recent published studies demonstrated that *TP53, ESR1, KMT2C, AKT1, PTEN*, and *NF1* were more frequently altered in metastatic HR+/HER2− breast cancer compared with the early ones, in accordance with a previous study, indicating their driving role in breast cancer metastasis and relapse (3, 47). Activating mutations of *ESR1* are common mechanisms of endocrine therapy resistance in HR +/HER2- advanced patients who exhibited responsiveness to selective ER degraders (48). Therefore, the detection of *ESR1* mutations is vital in tailoring effective therapeutic strategies for HR+/HER2- advanced patients.

Consistent with a previous study, breast cancer was associated with a high frequency of mutations in the p53 (72%), RTK-RAS (66.4%), PI3K (54.4%), and cell cycle (28.8%) signaling pathways. Among the genes involved these signaling pathways, *ABL1*, *FGFR1*, *CCND1*, *CDKN2A*, *ATM*, and *FGF3/4/19* were detected more frequently in R/M sites than primary tumors. The *CCND1* and *FGF3/4/19* genes are located in adjacent regions in the chromosome 11q13 region. The amplification of the chromosome 11q13 region is often observed in HR+ breast cancers and associated with poor prognosis and treatment failure (49–51). Genomic aberrations of *FGFR1* were composed of gene amplification, activating mutations, and gene fusions. Similar to a previous study (43), *FGFR1* gene amplification was identified in approximately 15% of patients with ER+ breast cancer. An *in vitro* study showed that high nuclear FGFR1 expression promotes antiestrogen resistance in ER+ primary tumors (52). More clinical studies are needed to explore the role of FGFR1 overexpression/amplification in estrogen sensitivity in ER+ breast cancer. The comparison of genes involved in common pathways between primary and R/M tumors may help to find the driving mutations accounting for treatment failure, metastasis, and relapse in breast cancer.

A copy number change at 9p24.1, which covered the loci for both *PD-L1* and *CD274*, was a potential biomarker of ICI response (53). Amplification of *PD-L1* has recently been evaluated in a pan-cancer analysis of 48,782 tumors, exhibiting a prevalence of 0.7% across tumors (54). In the present study, we found that *PD-L1* amplifications were more likely to be observed in R/M sites in TNBC breast cancer. A similar finding was also obtained in a previous study involving 5,399 cases from MSK-IMPACT and TCGA, in which the incidence of 9p24.1 amplifications was 1.0% and showed a significantly higher incidence in TNBC (55). Another study showed that *PD-L1* amplification may lead to increased PD-L1 expression *in vitro* (56). The predictive value of *PD-L1* amplifications in immunotherapy has been evaluated in samples of patients with metastatic breast cancer included in the randomized phase II SAFIR02-IMMUNO study, where patients with TNBC had a higher proportion of *PD-L1* amplifications and showed an improvement in OS with durvalumab in *PD-L1*-amplified tumors (hazard ratio = 0.17, 95% CI 0.05–0.55) (57). Recent research on 1,050 urothelial carcinoma cases showed nine tumors with *PD-L1* CN gain and nine tumors with *PD-L1* CN loss. Patients whose tumors harbored *PD-L1* amplification benefited from immunotherapy, while patients whose tumors had *PD-L1* CN loss experienced disease progression (58). A case report showed a urothelial carcinoma patient with *PD-L2* amplification experienced durable stable disease on pembrolizumab (59). A recent study conducted a longitudinal analysis of PD-L1 expression in surgical samples and recurrent biopsy in non-small cell lung cancer, revealing that PD-L1 expression exhibited dynamic changes during the course of the disease (60). Similar variations of PD-L1 were observed in breast cancer, with approximately one-third of patients showing discrepant PD-L1 expression between primary tumors and matched distant metastases (61). Therefore, it is necessary to re-evaluate the PD-L1 status of recurrent lesions to optimize immunotherapy strategies.

A comparison of the mutation spectrum by metastatic location demonstrated chest wall metastases more frequently harbored alterations in *FGFR1*, while liver metastases more frequently had mutations in *CCND1* and *FGF3/4/19*. In the MSKCC dataset, the *CCND1* and *FGF3/4/19* genes were the top 5 CNVs in liver, bone, lymph node, chest wall, and lung metastasis sites (42). The alteration rate of *FGFR1* amplification in tumors from the chest wall was lower than in tumors from other metastases, and no significant differences existed among metastases. The *ATRX* mutation rate was found to be lower in the whole MSKCC cohort. The disparity of the mutation frequencies of the above genes between our cohort and the MSKCC dataset may result from the small number of metastatic tumors involved in our study and the different mutation spectrums due to ethnicity. Further clinical exploration of genomic characteristics by metastatic location is needed in Chinese breast cancer patients.

There are also several limitations in this study. First, this study is a retrospective study that may suffer from selection bias (e.g., different molecular subtypes, metastatic locations). Moreover, a relatively small number of tumors originated from different metastatic locations; thus, some potentially valuable alterations may have been overlooked. Combined and comprehensive NGS and an examination of certain proteins, treatment regimens, and survival analyses may need to be undertaken in the future.

In conclusion, this study revealed the mutational features of primary and R/M tumors in Chinese patients with breast cancer. Our study identified mutational features and genomic signatures of primary and R/M tumors in three subtypes of breast cancers, which may be explored as potential therapeutic targets in our population. Moreover, the enrichment of *PD-L1* gene amplification in metastatic TNBC indicates the necessity to re-biopsy metastatic tumors for immunotherapy.

## Data Availability

The raw data supporting the conclusions of this article will be made available by the authors, without undue reservation.
