# Supplementary material for: The evolving landscape of genetic biomarkers for immunotherapy in primary and metastatic breast cancer
Source: Front Oncol. 2025 Mar 13;15:1522262. doi: 10.3389/fonc.2025.1522262 (PMC11966456; doi:10.3389/fonc.2025.1522262)
Supplement: Supplementary file 1 [file Table1.docx]

**Supplementary Table 1 The comparison of patient characteristics between our and MSKCC cohorts.**

|  |  |  |  |  |  |  |
| --- | --- | --- | --- | --- | --- | --- |
| **Variables** | | **Our cohort (n=563)** | | **MSKCC (n = 1756)** | | ***P*** |
|  |  | **No.** | **(%)** | **No.** | **(%)** |  |
| Age | |  |  |  |  | ＜0.001 |
| ≤50 years | | 386 | 68.6% | 802 | 46% |  |
| >50 years | | 177 | 31.4% | 954 | 54% |  |
| Sex | |  |  |  |  | 0.132 |
| Female | | 563 | 100% | 1746 | 99% |  |
| Male | | 0 | 0 | 10 | 1% |  |
| TNM stage | |  |  |  |  | 0.469 |
| I–II | | 325 | 57.7% | 1029 | 59% |  |
| III–IV | | 232 | 41.2% | 714 | 41% |  |
| Unknown | | 6 | 1.1% | 13 | 1% |  |

**Supplementary Table 2 The molecular subtypes of samples between our and MSKCC cohorts.**

|  | **Our cohort** | | | **MSKCC** | |
| --- | --- | --- | --- | --- | --- |
| Molecular subtypes | Primary (n=361) | Metastasis (n=229) | | Primary (n=918) | Metastasis (n=1000) |
| HR+/HER2- | 125(34.6%) | | 90(39.3%) | 719(78.3%) | 679(67.9%) |
| HER2+ | 79(21.9%) | | 49(21.4%) | 84(9.2%) | 145(14.5%) |
| TNBC | 157(43.5%) | | 90(39.3%) | 90(9.8%) | 81(8.1%) |
| Unknown | 0 | | 0 | 25(2.7) | 95(9.5%) |
